# Utility of the Enzyme-Linked Immunospot Interferon-γ–Release Assay to Predict the Risk of Cytomegalovirus Infection in Hematopoietic Cell Transplant Recipients

**DOI:** 10.1093/infdis/jiw064

**Published:** 2016-02-11

**Authors:** Lior Nesher, Dimpy P. Shah, Ella J. Ariza-Heredia, Jacques M. Azzi, Hala K. Siddiqui, Shasank S. Ghantoji, Lisa Y. Marsh, Lamprinos Michailidis, George Makedonas, Katy Rezvani, Elizabeth J. Shpall, Roy F. Chemaly

**Affiliations:** 1 Department of Infectious Diseases, Infection Control, and Employee Health; 2 Department of Stem Cell Transplantation and Cellular Therapy, University of Texas MD Anderson Cancer Center; 3 Center for Human Immunobiology, Department of Pediatrics, Baylor College of Medicine, Houston, Texas

**Keywords:** cytomegalovirus, ELISPOT, stem cell transplant, IGRA, bone marrow transplant

## Abstract

The ability to distinguish allogeneic hematopoietic cell transplant (allo-HCT) recipients at risk for cytomegalovirus (CMV) reactivation from those who are not is central for optimal CMV management strategies. Interferon γ (IFN-γ) produced by CMV-challenged T cells may serve as an immune marker differentiating these 2 populations. We prospectively monitored 63 CMV-seropositive allo-HCT recipients with a CMV-specific enzyme-linked immunospot (ELISPOT) assay and for CMV infection from the period before transplantation to day 100 after transplantation. Assay results above certain thresholds (50 spots per 250 000 cells for immediate early 1 or 100 spots per 250 000 cells for phosphoprotein 65) identified patients who were protected against CMV infection as long as they had no graft-versus-host disease and/or were not receiving systemic corticosteroids. Based on the multivariable Cox proportional hazards regression model, the only significant factor for preventing CMV reactivation was a CMV-specific ELISPOT response above the determined thresholds (adjusted hazard ratio, 0.21; 95% confidence interval, .05–.97; *P* = .046). Use of this assay as an additional tool for managing allo-HCT recipients at risk for CMV reactivation needs further validation in future studies. Application of this new approach may reduce the duration and intensity of CMV monitoring and the duration of prophylaxis or treatment with antiviral agents in those who have achieved CMV-specific immune reconstitution.

Cytomegalovirus (CMV) is a common cause of significant morbidity and mortality in patients with impaired cellular immunity, including patients with hematologic malignancies and recipients of solid organ and allogeneic hematopoietic cell transplants (allo-HCTs) [1–3]. Like most other herpesviruses, CMV becomes clinically quiescent after primary infection is brought under control by the host immune response, which usually occurs in childhood. The virus is known to possess immunomodulation abilities, enabling it to persist in a state of cellular latency. In this state, the infected cells either do not produce infectious virus or they allow the replication of virus at a very low rate, undetected by current methods, but retain the complete genome and have the potential to produce infective viruses at a later time [1, 4].

In immunocompromised hosts, the production and replication of CMV may lead to infection and a variety of end-organ diseases, such as retinitis in patients with AIDS and pneumonitis in HCT recipients. CMV has also been associated with rejection after solid organ transplantation [5, 6] and with graft-versus-host disease (GvHD) following allo-HCT [7–9]. Importantly, secondary bacterial and fungal infections occur frequently after CMV infection and, together with GvHD, are considered indirect effects of this immune-modulating virus [10].

In allo-HCT recipients, prophylaxis and/or preemptive therapy have been used to manage CMV infection. Owing to the toxic effects of the antiviral agents presently available, the prophylactic approach has fallen out of favor for a preemptive strategy. Despite the lack of a well-established, validated CMV load or antigenemia threshold for the initiation of therapy, the preemptive strategy is currently the most widely accepted practice for preventing CMV-associated end-organ disease after allo-HCT [1, 3, 11].

Cell-mediated immunity is considered the primary defense mechanism for controlling CMV replication [12]. Both CD4^+^ and CD8^+^ T cells are implicated in the protection against CMV infection; complex interactions involving a CD8^+^ T-cell response produce interferon γ (IFN-γ) and a host of other cytokines in response to the presence of CMV [13]. HCT recipients lacking CMV-specific CD8^+^ T cells develop CMV infection more frequently than those who have these cells, and reconstitution of CD8^+^ T-cell responses correlates with protection against CMV [14, 15]. IFN-γ has been shown to have a pivotal role in controlling CMV infection by the host's immune system [16]. The release of IFN-γ can be measured and IFN-γ may serve as an adaptive immune marker. A new enzyme-linked immunospot (ELISPOT)–based assay (T-SPOT.CMV assay; Oxford Immunotec, Oxfordshire, United Kingdom) measures IFN-γ levels in peripheral blood mononuclear cells and has recently become commercially available in the United States and Europe. It allows the assessment of T-cell immune activity by detecting the production of IFN-γ following ex vivo stimulation with CMV antigens (immediate early 1 [IE-1] and phosphoprotein 65 [pp65]); the peptides are designed to target both CD4^+^ and CD8^+^ T cells. Patients infected with CMV have T cells that recognize these antigens and secrete IFN-γ in response; the detection of individual IFN-γ–producing cells is the basis of the assay and allows the assessment of the CMV-specific activity of the immune system.

One of the major challenges in treating allo-HCT recipients is the ability to distinguish patients at high risk for CMV reactivation who require close monitoring from those who have achieved a level of immune reconstitution sufficiently adequate to control latent CMV from reactivating. We hypothesize that the IFN-γ produced by CMV-challenged T cells can serve as an adaptive immune marker differentiating these 2 populations. This study is a proof-of-concept study to assess the ability of the CMV-specific ELISPOT assay to identify CMV-seropositive allo-HCT recipients who have achieved immune reconstitution and so are not at risk for CMV reactivation.

## METHODS

For this prospective cohort study, conducted between October 2013 and June 2014, we enrolled CMV immunoglobulin–positive adult patients with leukemia who were in remission before they underwent allo-HCT. Patients were serially monitored until day 100 after transplantation via the CMV-specific ELISPOT assay and for CMV reactivation. Written informed consent was obtained from each participant, and all data collection and laboratory procedures were approved by the University of Texas MD Anderson Cancer Center Institution Review Board.

### Patient Management

CMV infection was managed in patients according to the standard of care at our institution, which consists of the preemptive strategy previously described [3]. Briefly, the preemptive strategy consists of antiviral therapy initiated upon CMV detection via weekly blood testing, using CMV antigenemia analysis. As per our institutional guidelines, antiviral therapy is recommended in all high-risk HCT recipients (ie, haplo-identical transplant recipients, cord blood transplant recipients, patients with GvHD, and/or patients receiving a prednisolone equivalent dose of ≥1 mg/kg) for any positive CMV antigenemia assays. For the remaining HCT recipients, who were considered at low risk, antiviral therapy was recommended for those with a CMV antigenemia level of ≥5 cells per million white blood cells. All patients received standardized GvHD prophylaxis in accordance with institutional protocols. Briefly, recipients of matched-related or matched-unrelated donor specimens received tacrolimus and methotrexate. Recipients of cord blood transplants with a myeloablative conditioning regimen received tacrolimus and mycophenolate mofetil, whereas those with a reduced-intensity or nonmyeloablative conditioning regimen received thymoglobulin.

### Data Collection

We collected data on various clinical characteristics, such as age, sex, race, CMV serostatus, type of transplant and conditioning regimen, results of routine laboratory tests (ie, complete blood count, including absolute lymphocyte count and absolute neutrophil count, and routine blood chemistry analyses, including liver function tests), and all positive microbiological data, including CMV antigenemia and blood culture findings. In addition, we also collected data on GvHD; immunosuppressive therapy; anti-CMV antiviral therapy, including prophylaxis and/or treatment; and work-ups related to possible CMV disease, such as computed tomography findings and bronchoscopic, colonoscopic, and ophthalmic examinations.

### Definitions

The primary end point of the study was the first episode of significant CMV reactivation, defined as the detection of CMV via the antigenemia assay in the patient's blood, after which anti-CMV therapy was initiated by the treating physician in accordance with our institutional guidelines. The secondary end point was CMV-associated end-organ disease as defined by Ljungman et al [2].

### Laboratory Analyses

Blood specimens were collected at 4 consecutive time points: before the initiation of the transplant conditioning regimen (pre-HCT) and day 30 (±7 days), day 60 (±7 days), and day 100 (±14 days) after transplantation. The samples were shipped on the same day as collection to Oxford Diagnostic Laboratories (Memphis, Tennessee), where the CMV-specific ELISPOT assay was performed within 32 hours of blood specimen collection, in accordance with the validated test procedures. When the cell count ranged from 75 000 to <250 000 cells per well, spot counts for both IE-1 and pp65 antigens were standardized by multiplying the spot counts by a correction factor to adjust to the optimal cell count of 250 000 cells per well. If the cell counts were <75 000 cells per well, the results were excluded from the analyses because of an insufficient cell count. In addition, nil control spot counts were subtracted from the spots count obtained for IE-1 and pp65 if the nil control was ≤10, and the sample results were excluded from analyses if the nil control was >10. The laboratory personnel were blinded to the patients' clinical information to minimize potential bias. The results of the assay were reported in batches to the research team; however, they were not available to the treating physicians and thus did not affect treatment decisions.

### Statistical Analyses

The first episode of significant CMV reactivation was the primary outcome of interest for these analyses. Clinical characteristics of allo-HCT recipients who did and those who did not develop significant CMV reactivation during the study period were compared using χ^2^ or Fisher exact tests, for categorical variables, and Student *t* tests, for continuous variables. Bivariable Cox proportional hazards regression analysis was used to discretize the 2 continuous variables into binary variables to identify their thresholds for predicting subsequent significant CMV reactivation (50 spots for IE-1 and 100 spots for pp65 were identified as optimal cutoffs for the primary outcome). Further, patients were classified into 2 categories: those with a high response to the CMV-specific ELISPOT assay (if the level of any antigen was above the aforementioned threshold) and those with a low response to the CMV-specific ELISPOT assay (if levels of both antigens were below the aforementioned threshold). Kaplan–Meier failure curves were generated to demonstrate the difference in the probability of significant CMV reactivation between the patients with low and those with high responses, using values from both antigens in the CMV-specific ELISPOT assay performed before such reactivation. Finally, a multivariable Cox proportional hazards regression model was built to identify the effect of CMV-specific ELISPOT assay results on subsequent significant CMV reactivation, when adjusted for known risk factors. A 2-sided *P* value of .05 was considered statistically significant. All statistical analyses were performed using Stata, version 13.0 (StataCorp, College Station, Texas).

## RESULTS

### Patient Characteristics

**Figure 1.**
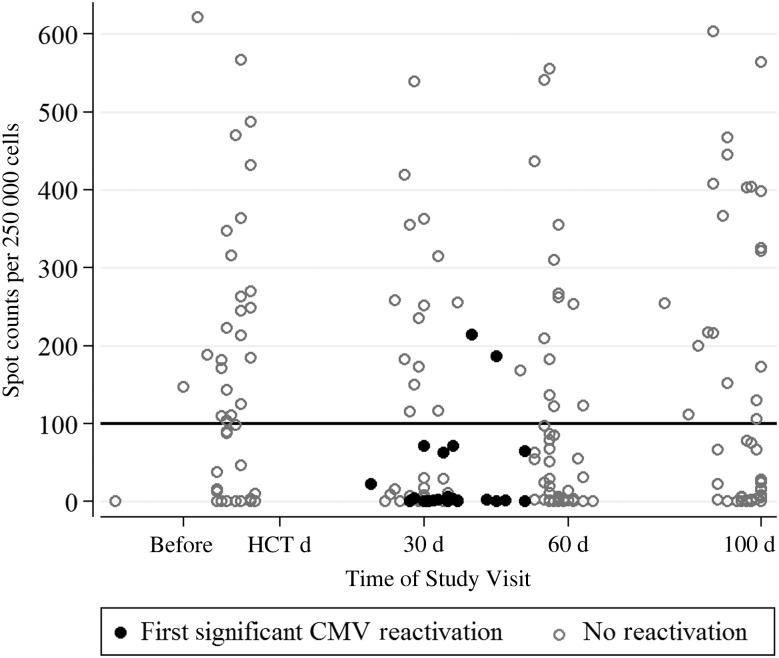
Scatterplot for cytomegalovirus (CMV) reactivation versus the number of spots produced in the CMV-specific enzyme-linked immunospot assay for phosphoprotein 65 antigen at various time points. The reference line at 100 spot counts denotes the study-specific threshold, which awaits confirmation by future studies. Abbreviation: HCT, hematopoietic cell transplantation.

Sixty-three patients with hematologic malignancies and CMV seropositivity were enrolled in this study and monitored for 100 days after allo-HCT. The majority of the required blood samples were collected at the predefined time points (day 30 – 98%; day 60 – 97%; day 100 – 94%). The majority of patients were white (78%), male (59%), and undergoing allo-HCT while in remission from acute leukemia (60%). More than half of the patients underwent HCT with a transplant from a matched-unrelated donor (56%), and most received a myeloablative conditioning regimen (94%). A total of 22 patients (35%) received hematopoietic cells from a CMV-seronegative donor, and 19 patients had developed GvHD and/or received corticosteroids for suspected or confirmed GvHD before CMV reactivation. Interestingly, the first episode of significant CMV reactivation (ie, de novo reactivation) occurred in 23 patient (37%) within the first 60 days after transplantation (mean time to de novo reactivation, 37 days; range, 19–56 days; Figure 1), with no de novo reactivation after day 60 in the remaining 40 patients who did not have a reactivation before day 60 (Figure 1 and Supplementary Figure 1). No statistically significant differences in baseline characteristics were observed between those who experienced significant CMV reactivation and those who did not (Table 1).

**Table 1.** Clinical Characteristics and Outcomes of Participants With and Those Without Cytomegalovirus (CMV) Reactivation Within 100 Days After Transplantation

Characteristic	Total (n = 63)	CMV Reactivation (n = 23)	No CMV Reactivation (n = 40)	*P* Value
Age, y	56 (21–73)	57 (21–69)	56 (24–73)	.494
Sex				.794
Male	37 (59)	14 (61)	23 (58)	
Female	26 (41)	9 (39)	17 (42)	
Race				.549
White	49 (78)	17 (74)	32 (80)	
African American	6 (10)	3 (13)	3 (7)	
Hispanic	7 (11)	2 (9)	5 (13)	
Asian	1 (2)	1 (4)	0	
Cancer type				.213
Acute leukemia	38 (60)	11 (48)	27 (68)	
Chronic leukemia	8 (13)	3 (13)	5 (12)	
Myelodysplastic syndrome	17 (27)	9 (39)	8 (20)	
Transplant source or type				.148
Match related donor	23 (37)	5 (22)	18 (45)	
Match unrelated donor	35 (56)	15 (65)	20 (50)	
Cord blood	5 (8)	3 (13)	2 (5)	
Stem cell source				.375
Peripheral	41 (65)	13 (57)	28 (70)	
Marrow	17 (27)	7 (30)	10 (25)	
Cord	5 (8)	3 (13)	2 (5)	
HCT donor CMV status				.280
CMV positive	41 (65)	13 (57)	28 (70)	
CMV negative	22 (35)	10 (43)	12 (30)	
Myeloablative conditioning regimen	58 (92)	19 (83)	39 (98)	.055
Time from HCT receipt to engraftment, mo, median (range)	12 (10–25)	12 (10–20)	12 (10–25)	.358
Corticosteroid^a^ use before reactivation	19 (31)	5 (22)	14 (36)	.208
GvHD prophylaxis regimen				
Tacrolimus	63 (100)	23 (100)	40 (100)	…
Methotrexate	58 (92)	20 (87)	38 (95)	.345
Mycophenolate	5 (8)	3 (13)	2 (5)	.345
GvHD^b^ (grade 1 or 2) before reactivation	12 (19)	4 (17)	8 (20)	.799
All-cause mortality	8 (13)	4 (17)	4 (10)	.318

Abbreviations: CMV, cytomegalovirus; GvHD, graft-versus-host disease; HCT, hematopoietic cell transplant.

^a^ Dose, >1 mg/kg.

^b^ Eight patients had skin GvHD, 2 with gastrointestinal GvHD, and 2 with both sites involved.

### CMV-Specific ELISPOT Assay

**Figure 2.**
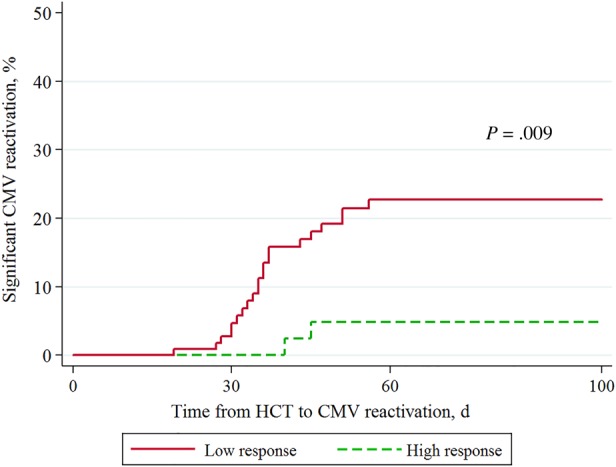
Kaplan–Meier failure curves of the time to significant cytomegalovirus (CMV) reactivation, stratified by high and low response to the CMV-specific enzyme-linked immunospot assay. Abbreviation: HCT, hematopoietic cell transplantation.

Serial assay responses over the study period are shown for those who had CMV reactivation (n = 23) and those who did not (n = 40; Figure 1). One pre-HCT assay result was discarded because the nil control was >10 and the result considered invalid. Among the 23 patients who had significant CMV reactivation, 21 had a low response (median spot counts, 0 for IE-1 antigen [range, 0–31] and 1 for pp65 antigen [range, 0–71]) in the preceding assay, whereas 2 patients with a high response (spot counts for IE-1 antigen were 11 and 208 and those for pp65 antigen were 186 and 214, respectively) had GvHD and were receiving systemic corticosteroids. None of the other patients with a high CMV-specific ELISPOT assay response had CMV reactivation. Kaplan–Meier failure estimates (Figure 2) showed significant differences in the CMV-specific ELISPOT assay response between patients who experienced significant CMV reactivation and those who did not (*P* = .009, by the log-rank test). Patients with a high response at day 30 after HCT (median spot counts, 32 for IE-1 antigen [range, 0–474] and 243 for pp65 antigen [range, 115–539]) had a lower chance of developing CMV reactivation than those with a low response (Figure 2). Based on multivariable Cox proportional hazards regression analysis, the only significant factor for preventing CMV reactivation was a high response at day 30 (adjusted hazard ratio, 0.21 [95% confidence interval, 0.05–0.97]; *P* = .046). Patients who received matched-unrelated donor or cord blood transplants were more likely to have reactivation than those who received a matched-related donor transplant; however, this factor was not significant after adjustment for other variables in the multivariable model. None of the other variables were significant predictors of CMV reactivation (Table 2).

**Table 2.** Multivariable Cox Proportional Hazards Regression Model for Risk Factors of the First Significant Cytomegalovirus (CMV) Reactivation

Variable	Crude HR (95% CI)	*P* Value	Adjusted HR (95% CI)	*P* Value
CMV-specific ELISPOT assay response^a^				
Low	1.00			
High	0.18 (0.04–0.78)	.022	0.21 (0.05–0.97)	.046
Age (per 10 y increase)	1.15 (0.84–1.56)	.384	1.1 (0.81–1.5)	.536
Sex				
Male	1.00		1.00	
Female	1.05 (0.45–2.43)	.907	0.81 (0.33–1.96)	.629
Transplant source/type				
MRD	1.00		1.00	
MUD/cord blood	2.22 (0.83–5.99)	.114	1.41 (0.49–4.09)	.523
Donor				
CMV seropositive	1.00		1.00	
CMV seronegative	1.34 (0.59–3.06)	.486	1.02 (0.44–2.39)	.963
Corticosteroid use before reactivation^b^				
No	1.00		1.00	
Yes	0.98 (0.96–1.01)	.132	0.98 (0.95–1.01)	.16
GvHD before reactivation^b^				
No	1.00		1.00	
Yes	0.99 (0.95–1.02)	.643	1.00 (0.97–1.04)	.777

Abbreviations: CI, confidence interval; ELISPOT, enzyme-linked immunospot; GvHD, graft-versus-host disease; HR, hazard ratio; MRD, matched related donor; MUD, matched unrelated donor.

^a^ A high response to the CMV-specific enzyme-linked immunospot assay indicates that either of the antigens had spot counts more than the study-specific thresholds of 50 spots for immediate early 1 or 100 spots for phosphoprotein 65. A low response in the CMV-specific ELISPOT assay indicates that both antigens had spot counts below the study-specific thresholds.

^b^ Time-varying covariates.

Interestingly, the 2 patients who had significant CMV reactivation despite a high response while receiving high-dose steroids and/or with GvHD sustained this high response up to day 100. One had 1 episode of significant CMV reactivation, whereas the other had 2 episodes within day 60. Both were alive at 1 year after transplantation. Of the remaining 21 patients who experienced significant CMV reactivation with a low response at day 30, 2 died within day 60, 1 developed a high response, and 9 of 18 (50%) with a persistent low response at day 60 had a second episode of CMV reactivation.

None of the patients developed CMV-associated end-organ disease, but 6 had a relapse of leukemia during the study period. A total of 8 patients (13%) died within 100 days after transplantation; however, none of the deaths were related to CMV.

### Diagnostic Accuracy

For the IE-1 antigen, the sensitivity for identifying allo-HCT patients at high risk for CMV reactivation in case of a low response was 96%, and the negative predictive value (NPV) denoting protection in case of a high response was 86%. For the pp65 antigen, the sensitivity for identifying allo-HCT patients at high risk for CMV reactivation in case of a low response was 91%, and the NPV denoting protection in case of high response was 88%. The overall accuracy for both antigens combined was similar (sensitivity, 91%; NPV, 88%).

## DISCUSSION

In this study, we evaluated the feasibility of the CMV-specific ELISPOT assay for measuring IFN-γ release as a marker of protection against CMV reactivation in patients with leukemia following allo-HCT. We observed that patients with a high CMV-specific ELISPOT assay response had no risk of developing significant CMV reactivation, compared with those with a low response, unless they had GvHD and/or were treated with systemic corticosteroids. These results support use of the CMV-specific ELISPOT assay to determine whether patients are protected against CMV reactivation following transplantation.

The major drawback of the preemptive approach is the uncertainty as to which patients remain at risk and require continuous monitoring and which patients have an immune system sufficiently reconstituted to control latent CMV infection, thereby obviating the need for further monitoring.

A more personalized laboratory-guided preventive strategy can be developed by precisely identifying the HCT recipient's immune protection against CMV reactivation. CMV-specific ELISPOT assay results above certain thresholds (50 spots for IE-1 and 100 spots for pp65) identified patients who, as long as they were not treated with corticosteroids, were protected against CMV infection, as seen in 2 patients who had a high CMV-specific ELISPOT assay response at day 30 but a week later developed GvHD, which required treatment with corticosteroids. Four days after the initiation of steroid therapy, these 2 patients developed CMV reactivation, indicating in our opinion that GvHD and/or steroids may negate the protective effect that was measured by the CMV-specific ELISPOT assay. We recommend repeating the CMV-specific ELISPOT assay for patient for whom a change a circumstances might reasonably be expected to have yielded diminished T-cell function since a prior test result. It is also conceivable that a lack of protection against CMV reactivation in the presence of CMV-specific T-cell response may be explained by innate immune deficiencies such as deficiency of natural killer cells [17] or failure to develop nonspecific natural killer cell cytotoxicity after allo-HCT [18].

HLA-restricted T-lymphocyte cytotoxic responses have also been associated with recovery from CMV infection in allo-HCT recipients [18]. Lower matrix protein pp65 is a dominant immune target for CMV-specific cytotoxic T lymphocytes (CTLs), and high frequencies of pp65-specific CTL precursors have been reported in CMV-seropositive individuals [19–21]. On the other hand, IE-1 is abundantly expressed earlier than pp65 during infection and continues to be expressed through the replicative cycle [22]. Hence, both of these antigens are indicative of the T-cell–mediated immune response against CMV infection in allo-HCT recipients. Using the most sensitive cutoffs for levels of IE-1 and pp65 antigens to create a binary variable for CMV-specific ELISPOT assay (high or low) response, we obtained very high sensitivity and NPVs for determining the risk for and protection from CMV reactivation early after transplantation, respectively. A previous study examining CMV-specific CTL responses in healthy individuals showed that, although neither protein alone was sufficient in all patients, all CMV-seropositive individuals mounted specific CTL responses against at least 1 of the proteins (IE-1 or pp65) [22]. Thus, the CMV-specific ELISPOT assay, which assesses the 2 most immunodominant CTL targets against CMV infection, would be an optimal assay to monitor the immune status of allo-HCT recipients for determining their protection from CMV infection after transplantation.

We demonstrated that allo-HCT recipients with higher numbers of CMV-specific IFN-γ–producing memory T cells were protected against CMV reactivation. This finding is consistent with other studies reporting similar clinical protection against CMV infection in both CMV-seropositive and CMV-seronegative kidney transplant recipients [23, 24]. In a recent study assessing the value of the CMV-specific enzyme-linked immunosorbent assay (ELISA; Qiagen, Valencia, California) in 43 allo-HCT recipients and 29 patients with hematologic malignancies, a high proportion of test results (33%) were indeterminate, likely because of the presence of lymphopenia during the early period after transplantation [25]. The authors reported that 29 patients developed CMV infection with negative CMV-specific ELISA results during DNAemia, of whom 17 had positive results of a subsequent ELISA, suggesting that there was agreement between CMV seropositivity and the CMV-specific ELISA results; however, the predictive value of this assay for determining the risk of CMV infection was not established [25]. Further studies are needed to compare the performance of the CMV-specific ELISPOT assays and ELISAs in predicting the risk of CMV reactivation in HCT recipients.

CMV-seropositive patients receiving allo-HCT from seronegative donors are at higher risk of developing CMV reactivation [26]. When adjusted for other factors, such as higher numbers of CMV-specific IFN-γ–producing memory T cells, which may provide adequate control of CMV replication, we did not observe a significantly higher rate of CMV reactivation in this group of patients, compared with those who received HCT from CMV-seropositive donors. This finding could be explained by the small sample size of our cohort. Donors with preformed anti-CMV immunoglobulin antibodies are more likely to have CTLs that are positive for multiple antigens; thus, it would be relevant to study the timing of the cell-mediated immune response of seronegative donors for CMV-seropositive recipients and its impact on conferring protection against CMV reactivation in this patient population. We did not evaluate the CMV-specific response in the donors, and this was one of the limitations of our study. Interestingly, we did not observe a significant association between GvHD and/or corticosteroids and CMV reactivation after adjustment for other risk factors (Table 2). This could be because of the small number of patients who had GvHD or were receiving corticosteroids in the cohort or because these 2 risk factors are surrogate markers of a low CMV T-cell response and were eliminated as confounders in the multivariate model. Other limitations include the small sample size of the cohort and the observational nature of a single center study; however, the results were reported after batch analysis to the research team, preventing bias in data collection and outcome ascertainment. The results of the assay were also not available to the treating physicians, thereby allowing for the observation of the course of this infection in patients who were treated according to an institutional-based preemptive therapy protocol without knowledge of the CMV-specific immunity status of the recipient. Thus, this new approach requires further validation in a multicenter study and measurement of the CMV load by PCR, with specific cutoffs as the primary outcome, because most, if not all, institutions have switched to molecular assays for CMV testing.

**Figure 3.**
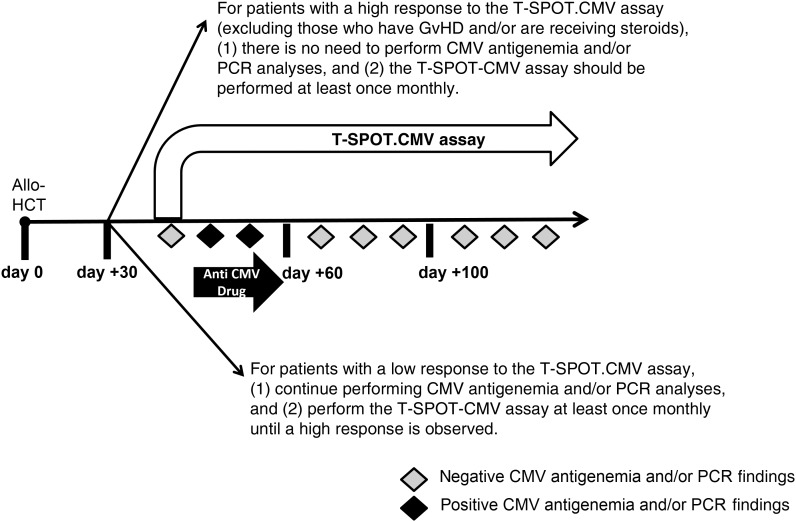
Use of cytomegalovirus (CMV)–specific enzyme-linked immunospot assay for developing an immune monitoring approach in allogeneic hematopoietic cell transplant (allo-HCT) recipients. Abbreviations: GvHD, graft-versus-host disease; PCR, polymerase chain reaction.

On the basis of our findings, we propose the examination of a new approach that includes immune monitoring for CMV in allo-HCT recipients during the posttransplantation period (Figure 3). This approach will evaluate the stimulus-dependent production of IFN-γ in samples obtained from patients by means of an IFN-γ release assay, to demonstrate when patients recover their immune system's ability to control latent CMV. We recommend testing with the CMV-specific ELISPOT assay at least once monthly in these patients, as well as repeat testing if there has been a major intervening event or treatment change that could affect the host's immune response against CMV; however, the optimal frequency of testing should be determined in further studies.

The overall benefit of the proposed immune-monitoring approach over the current preemptive strategy will be manifold. Application of this new approach may help reduce the duration and intensity of CMV monitoring and the duration of antiviral therapy or of prophylaxis, for those who have achieved CMV-specific immune reconstitution. In addition, and most importantly, the current preemptive strategy leads in many instances to excessive and prolonged duration of therapy among patients who might be able to spontaneously clear a low level of CMV reactivation if CMV-specific immune reconstitution is achieved. The ability of CMV-specific ELISPOT assay to identify patients who probably do not need therapy or further monitoring should be examined. Finally, the role of the CMV-specific ELISPOT assay response as a prognostic factor for clinical outcomes such as CMV-associated end-organ disease, graft outcome, and all-cause mortality in HCT recipients should be determined in future studies.

In conclusion, the CMV-specific ELISPOT assay appears to be an important diagnostic tool for identifying patients no longer at risk for CMV infection, following allo-HCT. The findings from this study should be confirmed in a multicenter trial.

## Supplementary Data

Supplementary materials are available at http://jid.oxfordjournals.org. Consisting of data provided by the author to benefit the reader, the posted materials are not copyedited and are the sole responsibility of the author, so questions or comments should be addressed to the author.

Supplementary Data
