# Supplementary material for: Utility of the Enzyme-Linked Immunospot Interferon-γ–Release Assay to Predict the Risk of Cytomegalovirus Infection in Hematopoietic Cell Transplant Recipients
Source: J Infect Dis. 2016 Feb 11;213(11):1701–7. doi: 10.1093/infdis/jiw064 (PMC4857477; doi:10.1093/infdis/jiw064)
Supplement: Supplementary Data [file supp_213_11_1701__index.html]

Utility of the Enzyme-Linked Immunospot Interferon-γ–Release Assay to Predict the Risk of Cytomegalovirus Infection in Hematopoietic Cell Transplant Recipients — Supplementary Data 

# Utility of the Enzyme-Linked Immunospot Interferon-γ–Release Assay to Predict the Risk of Cytomegalovirus Infection in Hematopoietic Cell Transplant Recipients

## Supplementary Data

Supplementary Data

- Supplementary Data - docx file
