# Supplementary material for: Utility of the Enzyme-Linked Immunospot Interferon-γ–Release Assay to Predict the Risk of Cytomegalovirus Infection in Hematopoietic Cell Transplant Recipients
Source: J Infect Dis. 2016 Feb 11;213(11):1701–7. doi: 10.1093/infdis/jiw064 (PMC4857477; doi:10.1093/infdis/jiw064)
Supplement: Supplementary Data [file supp_jiw064_jiw064supp_Data.docx]

**
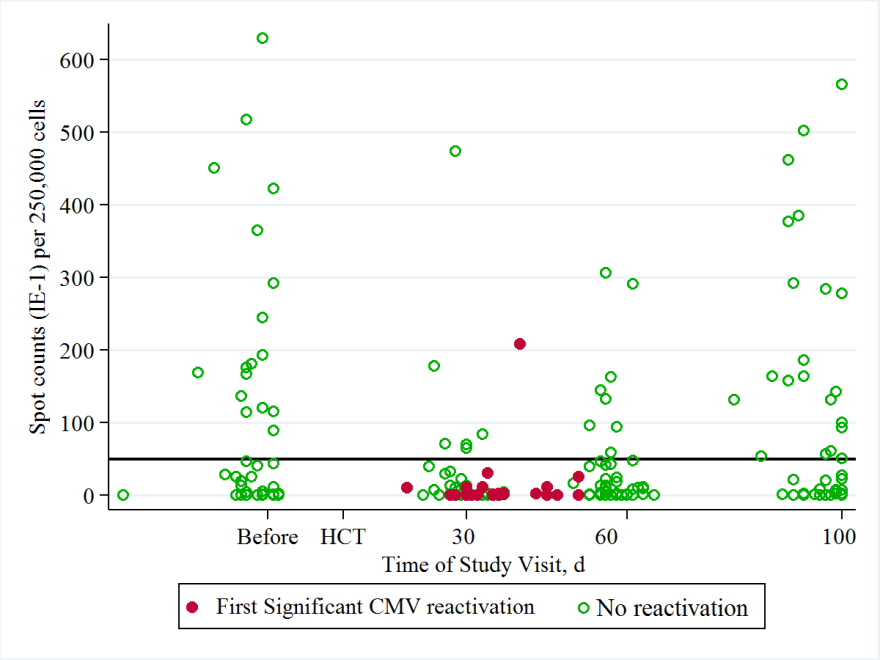
**

**Supplemental figure. Scatterplot for CMV reactivation versus number of spots produced in the CMV-specific ELISPOT assay for IE-1 antigen at various time points.**
